# ﻿A new species of the hitherto monospecific genus *Pleonoporus* Attems, 1938 (Diplopoda, Spirostreptida, Odontopygidae)

**DOI:** 10.3897/zookeys.1117.87765

**Published:** 2022-08-15

**Authors:** Henrik Enghoff, Nesrine Akkari

**Affiliations:** 1 Natural History Museum of Denmark, Universitetsparken 15, 2100 Copenhagen, Denmark Natural History Museum of Denmark Copenhagen Denmark; 2 Naturhistorisches Museum Wien, Burgring 7, 1010 Vienna, Austria Naturhistorisches Museum Wien Vienna Austria

**Keywords:** Africa, gonopods, natural history collections, new species, syntypes, taxonomy

## Abstract

The hitherto monospecific genus *Pleonoporus* is revised based on the syntypes of *P.robustus* Attems, 1938, housed in Naturhistorisches Museum Wien (NHMW), as well as on specimens of what we interpret as a new species of the genus, Pleonoporustanzanicus sp. nov., collected in Tanzania and housed in the Museum of Nature – Zoology, Leibnitz Institute for the Analysis of Biodiversity Change (ZMH) for more than a century. Both species are described based on habitus and gonopod structures and illustrated with micrographs, whereas scanning electron microscope images are also provided for the new species. This paper further highlights the importance of natural history collections for taxonomic research and studies on species diversity in general.

## ﻿Introduction

In his work on “Diplopoden des Belgischen Congo”, Attems (1938) continued with the colossal task of studying the myriapod diversity of the Congo, which he started almost a decade earlier (see also Attems 1929, 1934, 1935), aiming to document and describe the species of the area based on material sent to him for study by the Museum of the Belgian Congo (now The Royal Museum for Central Africa). In the same publication, Attems (1938) recorded 70 species and subspecies for the region, including 25 taxa new to science. Among these, 13 belonged to the family Odontopygidae (Diplopoda, Spirostreptida), classified in the genera *Odontopyge* Brandt, 1841; *Haplothysanus* Attems, 1909; *Chaleponcus* Attems, 1928; *Spinotarus* Attems, 1909; *Plethocrossus* Attems, 1909; *Rhamphidarpe* Attems, 1914; *Solenozophyllum* Attems, 1914 and *Pleonoporus* Attems, 1938. While some of these genera have recently been the subject of taxonomic revisions (e.g., Enghoff 2014, 2022), the genus *Pleonoporus* has remained known only from its original description and its only species *Pleonoporusrobustus* Attems, 1938, described from Elisabethville (now Lubumbashi) in the south-western part of the Democratic Republic of the Congo (Attems 1938). The species *Pleonoporusrobustus* has never been collected again, nor had its type series been re-studied. At the same time, no other representatives of the genus had ever been discovered.

In this work, we describe a new species of the genus *Pleonoporus*, *P.tanzanicus* sp. nov., based on material collected from Tanzania and housed in ZMH, and document the type species of the genus, *P.robustus*, based on the syntypes housed in NHMW. Both species are described, illustrated and compared, and the genus *Pleonoporus* is redefined.

## ﻿Material and methods

The specimens on which the new species is based were collected by “Ostafrika-Expedition der Hamburger Geographischen Gesellschaft in 1911.”

The syntypes of *Pleonoporusrobustus* were examined using a Nikon SMZ25 stereomicroscope and images were obtained with a Nikon DS-Ri2 camera mounted on the same stereomicroscope, using NIS-Elements Microscope Imaging Software (version 5.02) with an Extended Depth of Focus (EDF) patch (Nikon Corporation, Tokyo, Japan). A male of the new species was studied by scanning electron microscopy (SEM): body parts were cleaned with ultrasound, transferred to 96% ethanol, then to acetone, air dried, mounted on aluminium stubs or on triangles of flexible aluminium tape in turn mounted on a stub, coated with platinum/palladium and studied in a JEOL JSM-6335F scanning electron microscope. Images were processed in Adobe PhotoShop CS6 and assembled into figure plates with InDesign CS6 or Microsoft Publisher.

Morphological terminology mostly follows Enghoff (2022), with additional terms taken from Enghoff (2014).

Specimens are kept in the following collections:

**NHMD** Natural History Museum of Denmark

**NHMW** Naturhistorisches Museum Wien

**ZMH** Museum of Nature – Zoology, Leibnitz Institute for the Analysis of Biodiversity Change, Bonn

## ﻿Taxonomy

**Class Diplopoda De Blainville in Gervais, 1844**

**Order Spirostreptida Brandt, 1833**

**Family Odontopygidae Attems, 1909**

**Subfamily Archepyginae Manfredi, 1939**

### ﻿Tribe Prionopetalini Hoffman, 1991

#### ﻿Genus Pleonoporus

Taxon classificationAnimaliaSpirostreptidaOdontopygidae

Attems, 1938

38444CC4-B3A8-52A3-8BA0-30CEF945FE9C

##### Diagnosis.

Differs from all other genera of Archepyginae by having ozopores on body ring 5, a condition paralleled in the subfamily Peridontopyginae (see Enghoff 2022: 128). In other characters, especially the structure of the gonopod telomere, *Pleonoporus* resembles the genus *Spinotarsus* Attems, 1909.

##### Type species.

*Pleonoporusrobustus* Attems, 1938, by monotypy

##### Other included species.

*Pleonoporustanzanicus* sp. nov.

#### ﻿ Pleonoporus tanzanicus sp. nov.

Taxon classificationAnimaliaSpirostreptidaOdontopygidae

F4CFDAA1-4185-5721-851F-12921F69D060

https://zoobank.org/D825A23A-B618-41E9-B98B-0871CF67545B

Figs 1 , 2 , 3 , 4

##### Diagnosis.

Differs from *P.robustus*, its only congener, by having a long, slender, disto-laterad hooked spine on the gonopodcoxa; in *P.robustus*, there is no trace of such spine.

##### Material examined.

(total 5 ♂♂, 1 (juvenile) ♀). ***Holotype*.** Tanzania • ♂; Singida Region, Manyoni District, Kilimatinde; 05°50'S, 34°58'E; 26 Jan. – 08 Feb. 1911; E. Obst leg.; *verbatim* label text: “Ostafr.-Exp. d. Hamb. Geogr. Ges. Kilimatinde, Landschaft Ugogo Dr, E. Obst leg., 26.I –8.II. 1911. Geogr-. Ges. ded. 3.x.1912”; (ZMH) ***Paratypes*.** Tanzania • ♂; Singida Region, Manyoni District, Saranda-Sawa [here understood as the village of Saranda ca. 14 km N of Kilimatinde]; 05°43'S, 34°59'E; 15–16 Feb. 1911; E. Obst leg. ZMH-A0016675; NHMD 621850; *verbatim* label text: “Ostafr.-Exp. d. Hamb. Geogr. Ges. Ugogo und Turu, Saranda-Sawa, Dr. E. Obst leg., 15.–16.II. 1911, Geogr-. Ges. ded. 3.x.1912”; additional labels: “*Spinotarsus*?, R.L. Hoffman det. 1966”, “Odontopygidae: ?n.gen. oder viell. *Spinotarsus*?” [label by Krabbe?] • ♂; Singida Region, Manyoni District, Mahalala [here understood as the village of Muhalala ca. 11 km WNW of Kilimatinde]; 05°47'S, 34°53'E; 12–14 Feb. 1911; E. Obst leg.; ZMH-A0016676; *verbatim* label text: “Ostafr.-Exp. d. Hamb. Geogr. Ges. Mahalala, Landschaft Ugogo leg. Obst, 12/14.II. 1911” • 2 ♂♂, 1 (juvenile) ♀; same collection data as holotype, ZMH- A0016676; 1 ♂, same collection data as holotype, NHMW MY 10277 (♂).

##### Description.

(males). Size. Length ca. 8 cm. Diameter 5.5–6.0 mm. 60–62 podous rings, no apodous rings in front of telson.

**Figure 1.**
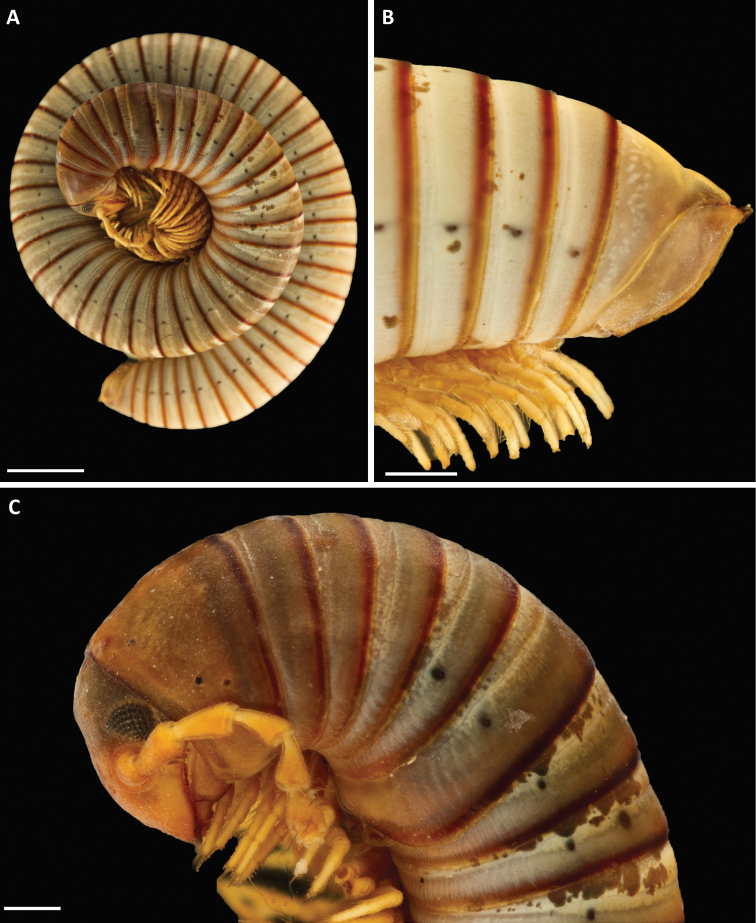
*Pleonoporustanzanicus* sp. nov. **A** paratype ♂, habitus (ZMHZMH-0016676) **B** holotype ♂ (ZMH-0016675) Last four body rings and telson **C** paratype ♂, head and first nine body rings (NHMW MY10277). Scale bars: 5 mm (**A**); 1 mm (**B, C**).

***Colour*** (Fig. 1). Quite faded after 111 years in alcohol. Head below antennal sockets, antennae and legs yellowish brown. Head above antennal sockets, collum, rings 2 and 6 almost uniformly dark brown. Other body rings greyish or whitish, with some irregular darker blotches; posterior part of metazonites amber. Traces of a pattern can be seen in some specimens, in which the prozonites from midway between the ozopore level and the midline are yellowish, white in the anterior half and a contrasting blackish in the posterior half; anterior part of body with traces of a narrower dorsal longitudinal light band flanked by a darker coloured zone.

***Head***. Area below supralabral setae vertically wrinkled, otherwise smooth; parietal furrow distinct, interocular furrow very faint; supralabral setae abraded and uncountable in most specimens, but six setae/sockets can be seen in some; eyes extending to medial tangent to antennal sockets. Mandibular stipes with a not very pronounced disto-ventral lobe; distal margin very shallowly emarginated.

***Collum***. With rectangular lateral lobes; a marginal furrow and one further furrow extending almost halfway to dorsal midline, two short furrows between them.

**Figure 2.**
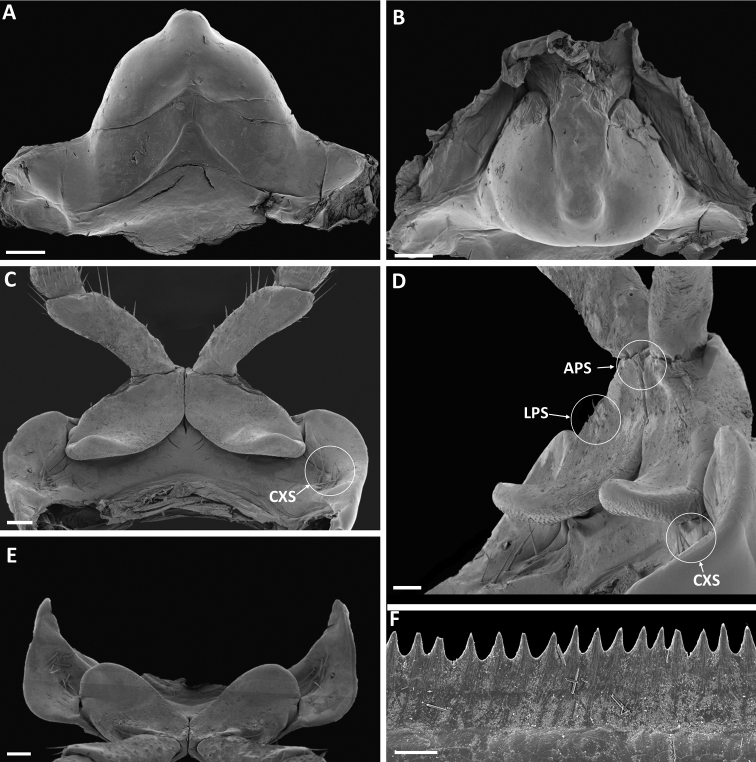
*Pleonoporustanzanicus* sp. nov., paratype, ♂ (NHMD 621850) **A, B** sternum 9 **A** anterior view **B** ventral view **C–E** first pair of legs **C** anterior view **D** sublateral view **E** ventral view **F** limbus. Abbreviations: *APS* = mesapical prefemoral setae; *CXS* = coxosternal setae; *LPS* = lateral prefemoral setae. Scale bars: 0.2 mm (**A–E**); 0.02 mm (**F**).

***Body rings***. Unvaulted; prozonites with fine, finely punctate ring furrows; suture straight, simple; metazonites with rather dense, deep longitudinal furrows in ventral part; on anterior body rings the furrows reach almost to ozopores level, further back they stop well below the pores and are not so deep. Ozopores starting on ring 5, visible as black dots (Fig. 1C). Limbus (Fig. 2F) with pointed, triangular lobes, lobes slightly longer than broad at base.

***Telson*** (Fig. 1B). Preanal ring not keeled. Anal valves smooth, with well-developed dorsal spine and raised margins; no ventral spine or “corner”; setae not visible, no ravelins. Subanal scale simple.

***Legs***. Length ca. 0.8 × body diameter. Prefemoral and tibial pads present on all legs from pair 6 backwards.

***First pair of legs*** (Fig. 2C–E). With short, broad, regularly rounded lobes, almost semicircular in ventral view. Ca. 6 long coxosternal setae (*CXS*) in a group lateral to, and well separated from, prefemoral lobe. Prefemora with two to three mesapical setae (*APS*) and ca. five lateral setae (*LPS*).

***Sternum 9*** (Fig. 2B, C). Massive; in anterior view triangular with a basal non-sclerotized incision resulting in an inverted V-shaped appearance; on posterior side strongly swollen.

**Figure 3.**
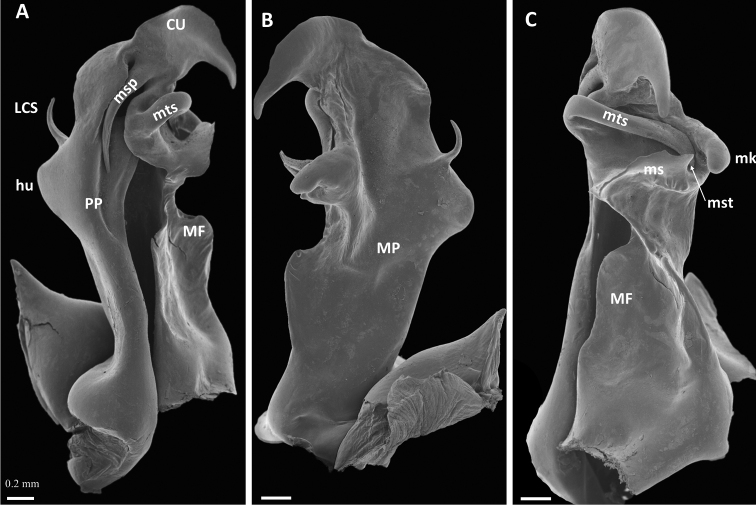
*Pleonoporustanzanicus* sp. nov., paratype, ♂ (NHMD 621850), left gonopodcoxa **A** anterior view **B** posterior view **C** mesal view. Abbreviations: *CU* = cucullus; *hu* = lateral hump; *LCS* = lateral coxal spine; *MF* = metaplical flange; *mk* = metaplical knob; *MP* = metaplica; *ms* = metaplical shelf; *msp* = metaplical spine; *mst* = metaplical shelf tooth; *mts* = metaplical transverse spine; *PP* = proplica. Scale bars: 0.2 mm.

***Gonopodcoxa*** (Fig. 3). In anterior view with a large hump (*hu*) on lateral margin; a long, slender, disto-laterad hooked spine (*LCS*) originating from distal part of hump. Mesal margin of proplica (*PP*) slightly sinuous, proplical lobe hidden from view by process (*msp*) from metaplica. Metaplica (*MP*) with large flange (*MF*) extending almost until half-height ofcoxa, ending abruptly but without a distal process. Metaplica distal to flange with a large horizontal shelf (*ms*) across mesal surface, ending posteriorly in triangular tooth (*mst*); a rounded knob (*mk*) projecting from mesal margin of metaplica, facing *mst*. Further distally, a lateral extension of the metaplica gives rise to a long, stout, transverse spine (*mts*) which curves posteriad across mesal surface ofcoxa, ending between *mst* and *mk*. Distal part ofcoxa, cucullus (*CU*) sensu Enghoff (2022), in anterior view resembling a bird’s head with the curved “beak” pointing meso-basad; a long, slender, slightly curved spine (*msp*) originating from base of cucullus on anterior side, extending basad along mesal margin of proplica.

**Figure 4.**
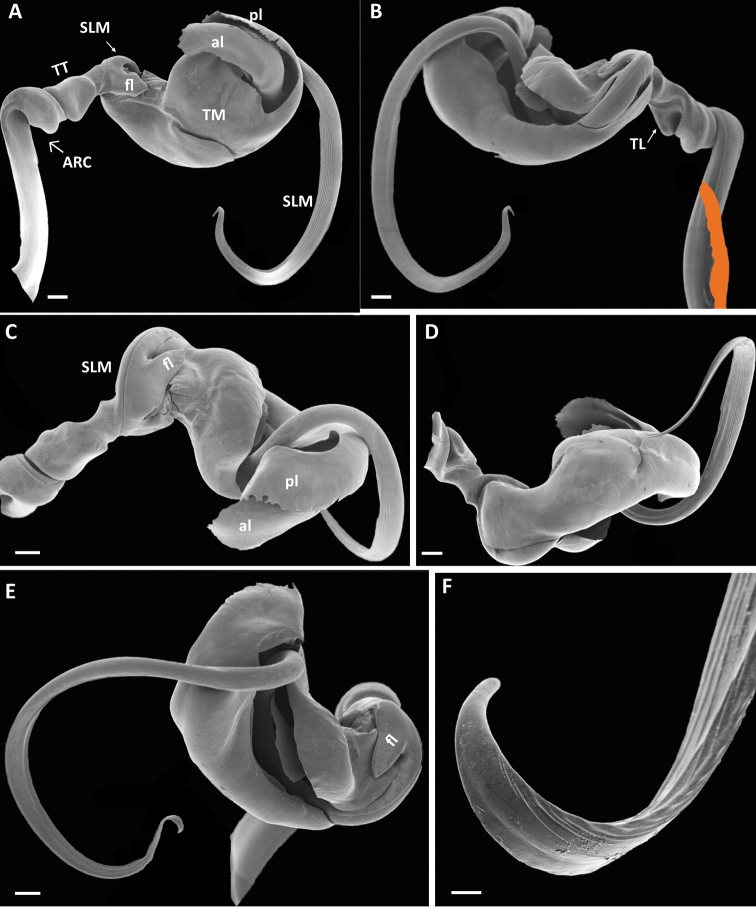
*Pleonoporustanzanicus* sp. n., paratype, ♂ (NHMD 621850), left gonopod telopodite**A** аnterior view **B** posterior view (basomere partly covered by mounting tape (orange)) **C** ventral (apical) view **D** basal (dorsal) view **E** mesal view **F** tip of solenomere. Abbreviations: *al* = anterior lobe of telomere; *ARC* = arculus; *fl* = basal flap of solenomere; *pl* = posterior lobe of telomere; *SLM* = solenomere; *TL I* = torsotope lobe; *TM* = telomere; *TT* = torsotope. Scale bars: 0.2 mm (**A–E**); 0.02 mm (**F**).

***Gonopodtelopodite*** (Fig. 4). Arculus (*ARC*) 90°; torsotope (*TT*) compact, with a rounded torsotope lobe (*TL*); no post-torsal spine. Solenomere (*SLM*) much longer than telomere, slender, whip-like, without a basal solenomeral spine, but with a small tongue-like flap (*fl*) at base; without any other process or modification. Telomere (*TM*) complex; basal shape a broad sheet curved in a semicircle and with the lateral margins partly folded in. Telomere distally separating into two lamellae, anterior lamella (*al*) with largely smooth margins, posterior lamella (*pl*) coarsely and irregularly serrate.

#### ﻿ Pleonoporus robustus

Taxon classificationAnimaliaSpirostreptidaOdontopygidae

Attems, 1938

F78F78F2-3FF2-55EE-9D5E-2FCE69C09154

Figs 5 , 6 , 7

##### Diagnosis.

Very similar to *Pleonoporustanzanicus* sp. nov., from which it differs by the absence of the lateral coxal spine (*LCS*) on the gonopods.

##### Material examined.

***Syntypes***: 6 ♂♂ (four of them broken in halves), 1♀ (NHMW MY 2666), one gonopod block (NHMW MY 9061), gonopods separated into left and right gonopod (NHMW MY 9062); 1 micro-preparation with fragment of a body ring; “Demokrat Republik Congo/Provinz Katanga, Elisabethville [Lubumbashi]/led. Denis A. 1935/don. Kong Mus./Attems 1937.”

##### Comments.

**Figure 5.**
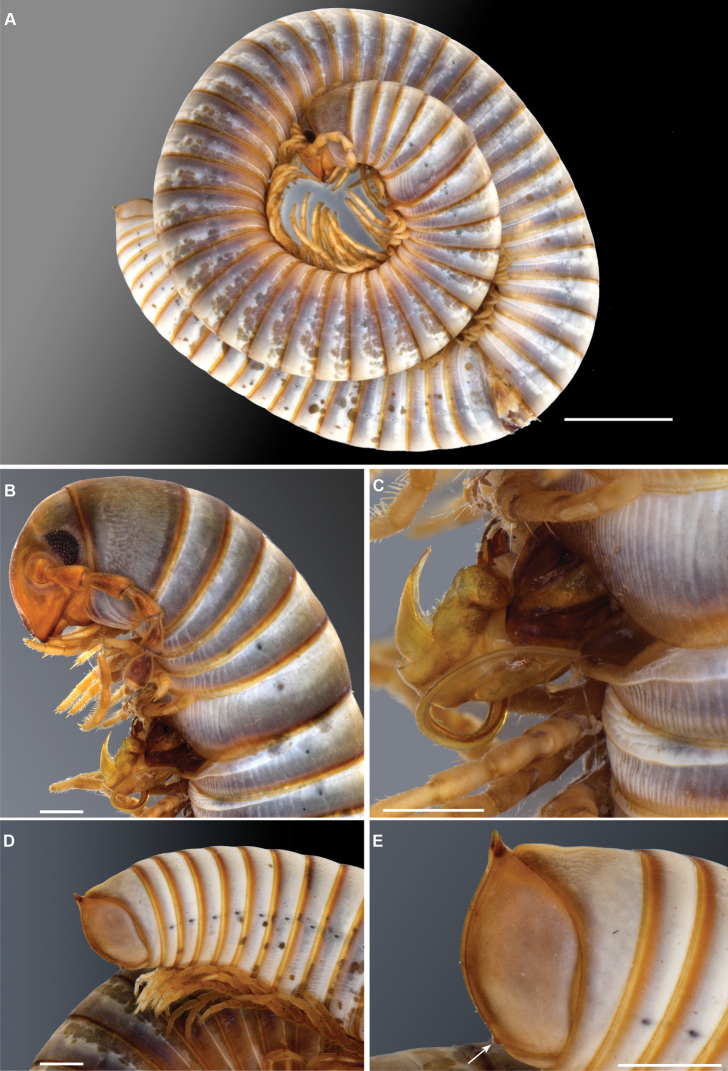
*Pleonoporusrobustus* Attems, 1938. Syntype ♂ (NHMW MY 2666) **A** male, in toto lateral view **B** head and anteriormost rings, lateral view **C** close-up of gonopod in situ, lateral view **D** posterior-most rings and telson, lateral view **E** close-up of the telson, arrow indicating ventral projections. Scale bars: 5 mm (**A**); 1 mm (**B–D**).

Attems (1938) mentioned a small projection on the ventral side of the anal valves “ein winziges Höckerchen” in his original description of the species. This structure was visible only in two of the male syntypes (Fig. 5E, arrow), whereas the rest of the specimens showed a regular ventral margin or just a slightly angular one. We dissected one of the males having anal valves with a regular ventral margin, and it showed gonopods identical to those described by Attems.

**Figure 6.**
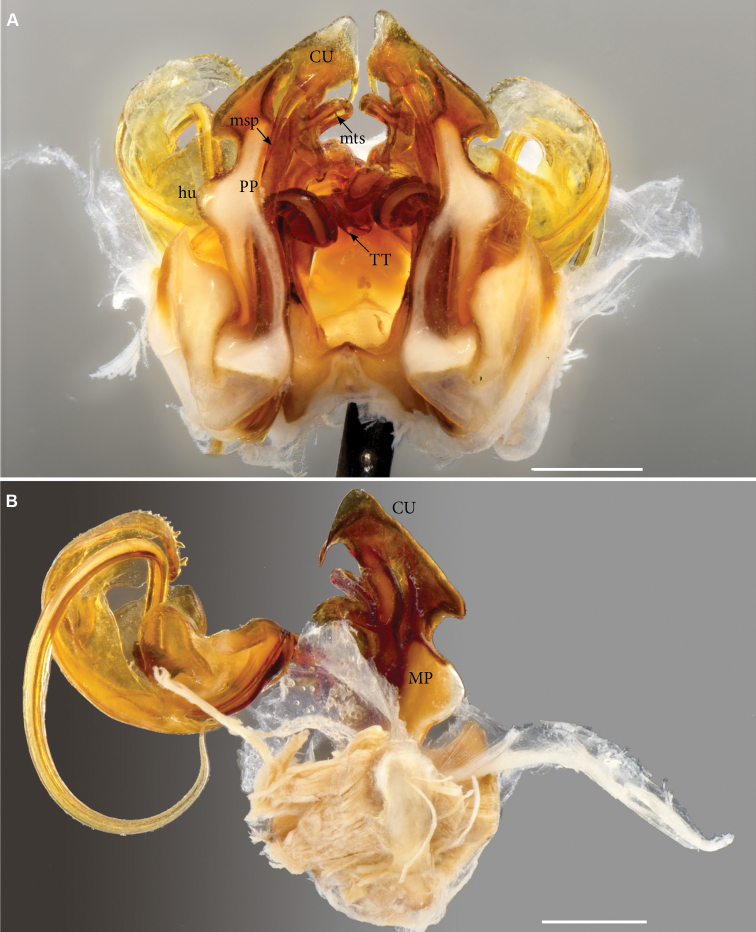
*Pleonoporusrobustus* Attems, 1938. Syntypes ♂ gonopods **A** gonopod block (NHMW MY9061), anterior view **B** left gonopod, posterior view (NHMW MY 9062). Abbreviations: *CU* = cucullus; *hu* = lateral hump; *MP* = metaplica; *msp* = metaplical spine; *mts* = metaplical transverse spine; *PP* = proplica; Scale bars: 1 mm.

**Figure 7.**
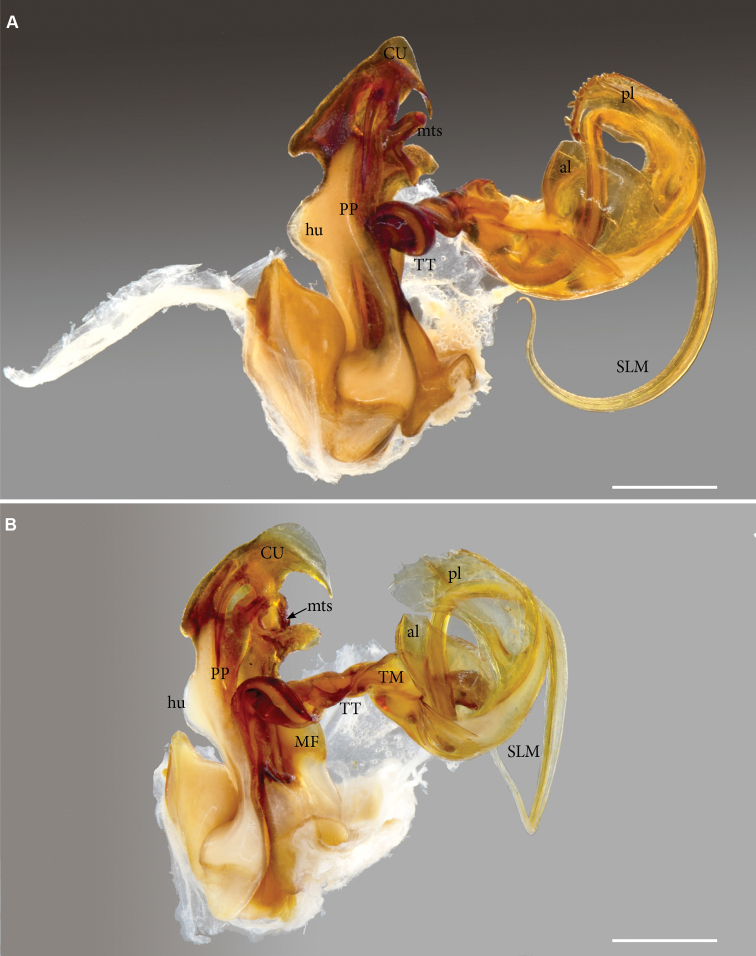
*Pleonoporusrobustus* Attems, 1938. Syntype ♂ gonopods (NHMW MY 9062) **A** right gonopod, anterior view **B** antero-mesal view. Abbreviations: al = anterior lobe of telomere, *CU* = cucullus; *hu* = lateral hump; *MF* = metaplical flange, *MP* = metaplica; *msp* = metaplical spine; *mts* = metaplical transverse spine; *PP* = proplica; pl = posterior lobe of telomere, *SLM* = solenomere; *TM* = telomere; *TT* = torsotope. Scale bars: 1 mm.

It was not possible to find the specimen from which Attems dissected and extracted the gonopods. However, the gonopod illustrations (Attems 1938: figs 86–89) perfectly match our illustrations (Figs 6–7) of the loose gonopods found in the jar, which we put in a separate vial (NHMW MY 9062). These may very well be the gonopods illustrated by Attems in the original description of the species.

## ﻿Discussion

The new species from Tanzania described here is very similar to *P.robustus* from the Democratic Republic of the Congo, but although there is really only one distinguishing character, i.e., the lateral coxal spine present in *P.tanzanicus* sp. nov. and absent in *P.robustus*, this spine is so conspicuous that we consider the Tanzanian specimens different from the Congolese ones at the species level. The geographical distance between the type localities of the two species is 1000 km, which in itself suggests (but does not prove) that different species are involved.

Another long-distance disjunction involving odontopygids was recorded by Hoffman and Howell (2012), although in this case the authors found no differences between type specimens of *Calyptomastixkakandae* (Kraus, 1958) from Upemba National Park in D.R. Congo and specimens referred to the same species but collected in SW Tanzania, some 600 km from the type locality. A similar distance separates the type locality of a third odontopygid, *Helicochetusmutaba* Kraus, 1960 (Kirungu, D.R. Congo), from two localities in the Iringa Region, Tanzania, from where *H.mutaba* was recorded by Enghoff (2018). The family Odontopygidae, in addition to containing a very large number of undescribed species, obviously also offers interesting distributional patterns.

Natural history collections continue to play a pivotal role as a source of undescribed species. A high number of unknown taxa are housed in museum collections, awaiting to be studied and described. An average shelf life of 20.7 years (range 0–206 years) was estimated for all species described in 2007 (Fontaine et al. 2012), and was explained by what these authors called the “taxonomic impediment”, referring to the shortage of taxonomists capable of identifying, describing and documenting species (Fontaine et al. 2012). The type specimens of *P.tanzanicus* sp. nov. were collected in 1911, and although at least one of them had already been studied by two specialists, they had a “shelf life” of 111 years before finally being described herein. Although this is less than the species *Ommatoiulusschubarti* Akkari & Enghoff, 2012 (149 years, see Akkari and Enghoff 2012), *P.tanzanicus* sp. nov. has largely exceeded the average estimated shelf life of undescribed taxa in museum collections.

In other instances, species have randomly been discovered when studying related taxa and recovered among the type material of another previously described species. The relatively recently described species *Annaminaattemsi* Golovatch, Geoffroy & Akkari 2017 is an example of such a species, which was described based on specimens mixed with the syntypes of *Annaminaxanthoptera* Attems, 1937 housed in NHMW and discovered by chance by one of us (NA) when studying that type series. The species *Haaseagruberi* Antić & Akkari, 2020 is another example of a species discovered when revising the genus based on the NHMW collection. The species had been misidentified as *Haaseaflavescens* (Latzel, 1884) by Attems in 1954, remained cryptic for some 70 years and was eventually described at the same time as a cavernicolous congener from Serbia (Antić and Akkari 2020). Misidenfications often happened in the past and in some cases discoveries of new taxa happened by comparing original species descriptions with subsequent literature related to these taxa. For example, Read (2005) noticed differences between the gonopods of *Cylindroiulusdistinctus* (Lucas, 1846) as illustrated in the original description (Lucas 1846) and those presented in the redescription of the species by Attems (1927) based on specimens collected in 1893 and housed in NHMW, the latter actually representing a completely new species, *Cylindroiulusattemsi* Read, 2005. Read (2005) based her description also on material from other natural history collections, with the oldest specimens housed in NHMD (then ZMUC) and dating back to 1869.

## Supplementary Material

XML Treatment for Pleonoporus

XML Treatment for Pleonoporus tanzanicus

XML Treatment for Pleonoporus robustus
